# Inconsistencies of genome annotations in apicomplexan parasites revealed by 5'-end-one-pass and full-length sequences of oligo-capped cDNAs

**DOI:** 10.1186/1471-2164-10-312

**Published:** 2009-07-15

**Authors:** Hiroyuki Wakaguri, Yutaka Suzuki, Masahide Sasaki, Sumio Sugano, Junichi Watanabe

**Affiliations:** 1 Department of Medical Genome Sciences, Graduate School of Frontier Sciences, The University of Tokyo, Kashiwanoha, Kashiwa, Chiba, Japan; 2 Departments of Parasitology, Institute of Medical Science, The University of Tokyo, Shirokanedai, Minatoku, Tokyo, Japan

## Abstract

**Background:**

Apicomplexan parasites are causative agents of various diseases including malaria and have been targets of extensive genomic sequencing. We generated 5'-EST collections for six apicomplexa parasites using our full-length oligo-capping cDNA library method. To improve upon the current genome annotations, as well as to validate the importance for physical cDNA clone resources, we generated a large-scale collection of full-length cDNAs for several apicomplexa parasites.

**Results:**

In this study, we used a total of 61,056 5'-end-single-pass cDNA sequences from *Plasmodium falciparum*, *P. vivax*, *P. yoelii*, *P. berghei*, *Cryptosporidium parvum*, and *Toxoplasma gondii*. We compared these partially sequenced cDNA sequences with the currently annotated gene models and observed significant inconsistencies between the two datasets. In particular, we found that on average 14% of the exons in the current gene models were not supported by any cDNA evidence, and that 16% of the current gene models may contain at least one mis-annotation and should be re-evaluated. We also identified a large number of transcripts that had been previously unidentified. For 732 cDNAs in *T. gondii*, the entire sequences were determined in order to evaluate the annotated gene models at the complete full-length transcript level. We found that 41% of the *T. gondii *gene models contained at least one inconsistency. We also identified and confirmed by RT-PCR 140 previously unidentified transcripts found in the intergenic regions of the current gene annotations. We show that the majority of these discrepancies are due to questionable predictions of one or two extra exons in the upstream or downstream regions of the genes.

**Conclusion:**

Our data indicates that the current gene models are likely to still be incomplete and have much room for improvement. Our unique full-length cDNA information is especially useful for further refinement of the annotations for the genomes of apicomplexa parasites.

## Background

Apicomplexa is a phylum of protozoan parasites that infects both humans and animals, causing serious health problems world-wide. *Plasmodium falciparum *(Pf) and *Plasmodium vivax *(Pv), for example, cause malaria, which kills over a million people every year [1,2]. *Toxoplasma gondii *(Tg) infects one third of the entire human population, causing brain and eye defects in the unborn fetuses of infected women [3]. *Cryptosporidium parvum *(Cp) infects humans and other warm-blooded animals, causing severe diarrhea [4]. Genome sequencing projects for at least 15 species of apicomplexa, including several *Plasmodium *species [5-7], two *Theileria *species [8,9], *Babesia bovis *[10], Cp [11] and Tg, have been carried out during the last decade.

The resulting genomic sequences have been analyzed, revealing that even though the apicomplexan parasites are believed to have been derived from a common ancestor, their genome sizes and compositions vary widely. The Cp genome is only 9.1 Mb, with only 5% of its genes containing introns, a proportion which nearly parallels that of the *Saccharomyces cerevisiae *genome [11]. The Tg genome, by contrast, is 65 Mb, averages 4.1 introns per gene, and has a G+C content of 52% [3]; whereas the Pf genome is 23 Mb, and is extremely A+T rich, having a G+C content of just 19% [7]. Respective genome information for each of these species has been made publicly available in one or more of the following databases: PlasmoDB [12-14], CryptoDB [15-17], ToxoDB [18,19], EuPathDB [20], and GeneDB [21].

Obviously, accurately annotated genomes are important tools for elucidating the genetic basis of parasiticism in apicomplexa. Such genetic knowledge will form the basis for drug development and potential vaccine candidates for these parasites. However, the quality of the accumulated genomic data is currently insufficient for these purposes. The genomic sequences of Py and Pb are still very incomplete, consisting of numerous short contigs (the N50 contig lengths of Py and Pb are only 7.7 kb and 2.8 kb, respectively; note the N50 size is the length such that 50% of the assembled genome lies in blocks of the N50 size or longer). An even more serious issue is that the genome-associated gene annotations (gene models) appear to be imperfect. Even for the well-annotated *P. falciparum *genome, recent reports have suggested it contains many errors [22]. Because the structures of the genomes and genes are very different from species to species, it is difficult to make precise, uniform gene prediction, using computational methods such as GENSCAN [23] or GlimmerM [24]. Therefore, experimental evidence, such as cDNA sequences, is extremely important and should be more intensively collected and taken into consideration for annotation purposes.

We previously developed a method, called oligo-capping, for constructing full-length cDNA libraries and have used it to collect full-length cDNAs from numerous organisms [25]. These cDNA sequences have been published online in two databases: Full-Parasites and Comparasite [26]. Full-Parasites [27] contains 5'-end-single-pass-read expressed-sequence-tags (5'-ESTs) for the Pf, Pv, Py, Pb, Cp and Tg genomes, and for the tapeworm *Echinococcus multilocularis *[28]. Comparasite [29] is an integrated database containing the transcriptomes of the same six apicomplexa species [26]. In it, homologous gene groups are clustered and any combination of these species can be comparatively analyzed. While analyzing the cDNA data in these databases, we noticed significant inconsistencies between our cDNA annotations and those of the publically available annotated genes.

In this study, we first analyzed 61,056 5'-end partially sequenced cDNAs which were isolated from six apicomplexan parasite full-length cDNA libraries. We found that a significant number of current gene models contain inconsistencies and therefore should be re-evaluated. To evaluate the gene models at the complete sequence level, we completely sequenced 732 full-length Tg cDNAs and drew the same conclusions. In addition, we found that the possible errors in the publically available annotations were largely due to overprediction of the exons. Here we report the first, large-scale systematic evaluation of the current genomic annotation of apicomplexan parasites based on our unique full-length cDNA data.

## Results and discussion

### Mapping and clustering of the 5'-ESTs from oligo-capped full-length cDNA libraries

We generated 5'-EST cDNA sequence collections from six apicomplexan parasites and mapped them to their respective genomic sequences (PlasmoDB pf_rel5.4, pv_rel5.4, py_rel5.4, pb_rel5.4, cp_rel3.7 and tg_rel4.3) using SIM4 [30]. We successfully mapped 7,313 cDNAs to the Pf genome, 7,686 to the Pv genome, 7,794 to the Py genome, 692 to the Pb genome, 9,656 to the Cp genome, and 6,218 to the Tg genome (Additional files 1 and 2; [DDBJ: DK887268–DK936566]). These data are also available at our websites [27,31].

The mapped cDNA sequences were clustered and compared with the annotated gene models in the PlasmoDB, CryptDB and ToxoDB databases [12,15,18]. An annotated gene model was considered to correspond to a cDNA when at least one of its exons overlapped the cDNA. The total number of cDNAs corresponding to annotated gene models was 6,383 for Pf, 6,349 for Pv, 7,429 for Py, 556 for Pb, 9,134 for Cp and 5,447 for Tg. Among the annotated gene models, 1,628 (29%) from Pf, 1,522 (28%) from Pv, 1,468 (19%) from Py, 277 (2%) from Pb, 673 (17%) from Cp, and 814 (11%) from Tg were represented in our cDNA collections. Conversely, 624 cDNA clusters in Pf, 701 in Pv, 256 in Py, 92 in Pb, 131 in Cp, and 390 in Tg did not correspond to any annotated gene model (Additional files 1 and 2). Although some of these latter clusters may actually be contaminated genomic DNA, we believe most, if not all, represent actual transcripts because; i) some are spliced (9% in Pf, 25% in Pv, 23% in Py, 10% in Pb, 15% in Cp and 13% in Tg) and definitely represent expressed genes; ii) RT-PCR analysis in Toxoplasma indicates that most of them are RNAs but not DNAs; and iii) intensive DNase treatment was performed during library construction.

### Discrepancies between the annotated gene models and our cDNAs

**Figure 1:**
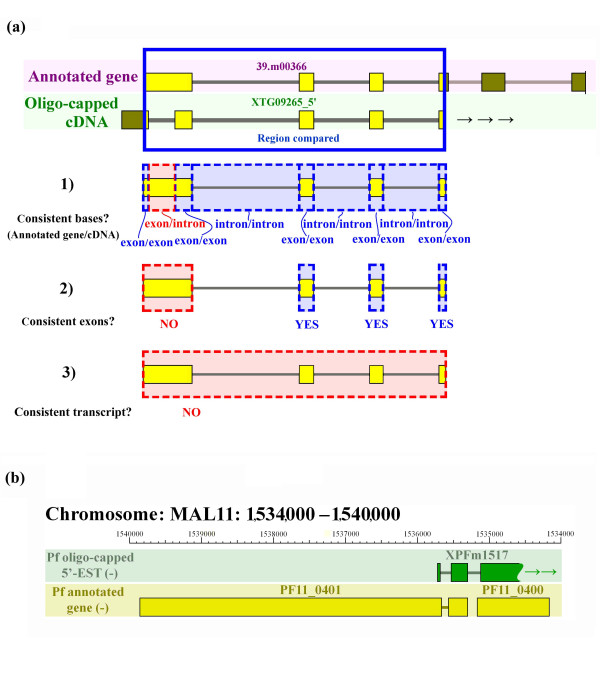
**Example of the evaluation of and merging of a 5'-end cDNA sequence with its annotated gene model**. (a) Genomic regions that were covered by both an annotated gene model and a cDNA were used for evaluation purposes (yellow boxes indicate exons, gray lines indicate introns). Inconsistency is illustrated here at the base level (1) and at the exon level (2). Blue dashed boxes and red dashed boxes represent consistent and inconsistent parts, respectively. Results of the evaluation are shown on the left. Because of the inconsistencies shown in (1) and (2), this annotated gene model was categorized as inconsistent at the transcript level in (3). (b) Example of a cDNA that corresponds to two annotated genes. The 5'-EST of the oligo-capped cDNA (XPFm1517; first line) and annotated gene models (PF11_0401 and PF11_0100; second line) are shown. XPFm1517 represents the three exons at the 5'-end, with an undetermined 3'-end.

Using the 5'-end partial cDNA sequences corresponding to the annotated gene models, we evaluated the inconsistencies between them at three levels (a typical example is shown in Figure 1a):

(1) Nucleotide-level: The total number of nucleotides either "exonic" in the cDNAs that are mis-annotated as "intronic" in the gene models or vice versa.

(2) Exon-level: The number of exons that contained at least one mis-annotated nucleotide resulting in one or more different exon-intron boundaries.

(3) Gene-level: The number of annotated gene models that contained at least one mis-annotated exon.

We found that the average error rates in the gene models of the apicomplexa genomes was (1) 4% at the nucleotide-level, resulting in (2) a 14% error rate at the exon-level and (3) a 16% error rate at the annotated gene-level (Table 1). We also found that some cDNA clusters (16 cases in Pf) corresponded to more than one annotated gene, with the two adjacent annotated genes likely representing erroneously separated genes that should be re-annotated as one gene (Figure 1b).

**Table 1:** Discrepancies between oligo-capped cDNAs and annotated gene models

Species	Nucleotide level (%)	Exon level Discrepant No./Total No. (%)	Gene level Discrepant No./Total No. (%)
Pf	2.6%	175/2,075 (8%)	133/1,543 (9%)
Pv	3.9%	320/2,371 (13%)	258/1,457 (18%)
Py	7.5%	302/1,939 (16%)	233/1,340 (17%)
Pb	3.0%	94/377 (25%)	53/254 (21%)
Cp	1.1%	33/669 (5%)	32/658 (5%)
Tg	7.0%	245/1,556 (16%)	191/780 (24%)

Average	4.2%	14%	16%

For further detailed information see the text.

There were also a significant number of un-annotated transcripts in each of the genomes. We found that 624 (28%) cDNA clusters in Pf were not represented by any of the annotated gene models. Similarly, 32% of the cDNA clusters in Pv, 15% in Py, 25% in Pb, 16% in Cp, and 32% in Tg were not previously annotated. It is possible that some of these clusters might actually overlap with annotated genes, once the entire sequences are determined, but a significant proportion of them appear to represent hitherto un-annotated genes (see section on non-overlapping cDNAs in Tg).

Our cDNA-based evaluation of the current genome annotations indicates that the current gene models are still incomplete and contain many errors. Because our evaluation is based only on the 5'-end of the transcripts, more inconsistencies will likely be revealed once the entire cDNA sequences are determined and evaluated (See section on completely sequenced cDNAs in Tg).

### Possible causes of the discrepancies

It was surprising that even the *P*. *falciparum *genome, which is the most intensively annotated apicomplexa genome to date [7,32,33], contains so many discrepancies. Although some experimental evidence, ranging from random EST sequencing to RT-PCR of individual genes had been collected, the amount seems to be insufficient. A more thorough accumulation of full-length cDNA information is necessary for more precise genome annotation of Pf.

It was not surprising to find far more inconsistencies for *T. gondii *(Tg) than Pf because of its larger genome size and more frequent use of introns, which makes gene prediction more difficult (Additional file 3). Indeed, the frequency (32%) of un-annotated cDNA clusters was the highest for Tg. In contrast, the genome annotations for Cp were exceptionally accurate because the Cp genome is very small and the transcripts rarely have introns (Additional file 3). Inaccurate gene annotations might be an intrinsic problem with genomes as complex as that of Tg.

Completeness of the genomic sequences is an additional factor in the correct annotation of open reading frames. The genome sequences of Py and Pb still consist of many small contigs. The number of annotated genes in Py and Pb are 7,861 and 12,235, respectively, both of which are considerably higher than the 5,490 annotated genes in Pf. Py and Pb are evolutionarily close, so the large difference in the number of annotated genes may reflect false annotations in both genomes. In fact, we observed that in Pb, 27% of the protein coding regions of the annotated genes did not start from an ATG site, so their CDSs are intrinsically incorrect. When we evaluated these annotations in more detail, we sometimes found that two neighboring annotated genes in Py or Pb that mapped onto different contigs were only, correctly, represented as one gene in Pf, leading to more genes having been annotated in Py and Pb than in Pf. Many such split transcripts could be fixed by considering our full-length cDNA information, thus helping to sew together the current patchwork of genomic contigs (Additional file 4).

The base composition of the genome seems to be less deterministic when it comes to discrepancies. For example, Plasmodium genomes are generally extremely A+T rich, with the Pf genome being the most biased and the Pv genome being the most moderate (G+C content of 19%, 42%, 23%, and 23% for Pf, Pv, Py, and Pb, respectively) [7]. In spite of the varying G+C content among these species and presumed conservation of most of the genes, the accuracy of the genomic annotation varied substantially (discrepancy at the gene-level of 9%, 18%, 17%, and 21% for Pf, Pv, Py, and Pb, respectively). The more accurate annotation of the Pf genome may have been achieved by more careful optimization of the computational protocols, such as the use of GlimmerM [24] along with more detailed manual inspection [32].

### Utilization of full-length cDNAs for identification of transcriptional start sites and 5'untranslated regions

The second advantage in using the 5'-ends of the full-length cDNA sequences was that we could annotate the exact transcriptional start sites (TSSs) and the complete 5' untranslated regions (5'-UTRs) of the transcripts. This information is essential for characterizing putative transcriptional regulatory elements in the upstream promoter regions and translational control elements embedded in the 5'-UTRs [34]. As almost all current gene annotations are "protein-coding sequence (CDS) based annotations", they are generally useless for analyzing regions upstream of the initiator ATG sequences. For example, in Table 2, in several cases we found that there were one or more introns between the predicted initiator ATG and the TSS, specifically 22% of the cases in Pf, 20% in Pv, 28% in Py, 22% in Pb, 3% in Cp, and 13% in Tg. Since no information on the TSSs or UTRs could be obtained from the proximal regions of the current gene models, they were not useful for an evaluation like this.

**Table 2:** Characteristic features of the 5'-UTRs

Species	Frequency of genes containing intron(s) in the 5'-UTRs (%)	Average 5'-UTR length (bp)	Standard deviation of 5'-UTR length (bp)
Pf	22%	303	155
Pv	20%	304	199
Py	28%	345	174
Pb	22%	299	166
Cp	3%	137	116
Tg	13%	288	172
			
Average	18%	279	164

See Methods for further details.

Tables 2 and 3 show the results for the statistical analyses of the 5'-UTRs and TSSs. The average 5'-UTR length was shortest for Cp, which may reflect the fact that both the genome size and the average gene size are smallest in this species. We also found that the TSSs of the *Plasmodium *species are much more dispersed than those of Tg or Cp. These characteristic features of the 5'-ends of the mRNAs may be a sign of differences in both transcriptional and translational control of gene expression among apicomplexan parasites. In addition, statistical analysis revealed that the 5'-UTR length of the genes associated with the Gene Ontology term "ribosome" is shorter than the other genes in both Tg and Py (p < 2e-12 and p < 5e-10, respectively (Additional file 5)); however, this tendency was not observed in the other species. The unique data presented here should serve as a firm foundation for future analysis of the transcriptional regulatory networks of parasite genes.

**Table 3:** Characteristic features of the TSSs

Species	Average number of cDNA members per cluster	Average number of TSS positions per cluster	Average of TSS standard deviation (bp)
Pf	7	5	80
Pv	7	4	61
Py	9	6	61
Pb	3	2	27
Cp	22	5	16
Tg	11	4	34

4th column: average standard deviation of fluctuating positions of TSSs for each cluster. For further information see Methods.

### Complete sequencing of full-length cDNAs in Toxoplasma

For a more detailed evaluation of the annotated gene models, we chose representative full-length Tg cDNA clones from each of 1,204 clusters and attempted to completely sequence them by the primer-walking method. Finally, we were able to determine 732 full-length sequences with an average length of 1.5 kb, as compared to 1.8 kb for the annotated transcripts, corresponding to the 1,204 clusters. These sequences included 592 cDNAs that overlapped one or more annotated genes (586 overlapped only a single annotated gene) and 140 cDNAs that did not overlap any annotated gene.

**Figure 2:**
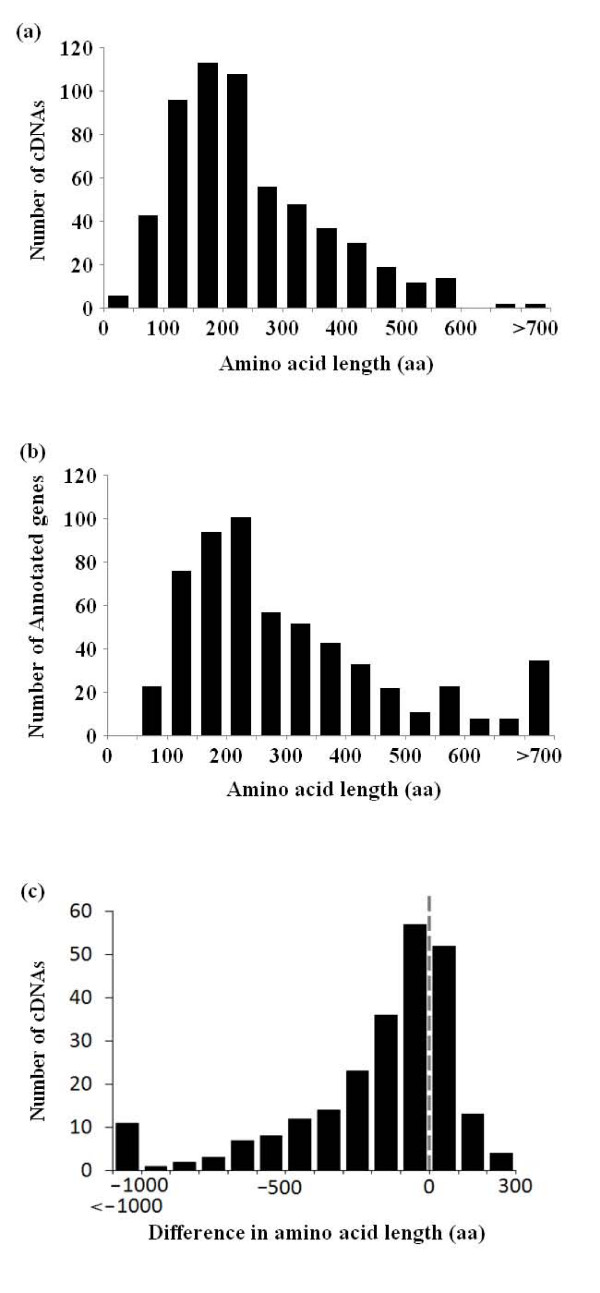
**Length distribution of the deduced amino acids**. Length distribution of the deduced amino acids in Toxoplasma full-length cDNAs. Length distribution of the deduced amino acids in annotated genes corresponding to full-length cDNAs. Length distribution of the inconsistent part of the amino acid sequences. Negative values on the horizontal axis indicate that the amino acid sequences were shorter in the cDNAs. Data for which there was no difference in amino acid length is not shown.

Based on the cDNA sequences, we assigned the longest ORFs as the putative protein coding sequences (CDSs). As shown in Figure 2, for cDNAs overlapping annotated genes, 580 of the deduced CDSs (99%) were longer than 150 bp (the shortest length for the annotated gene models) with an average length of 661 bp, suggesting that most of the full-length cDNAs we used for this analysis were indeed protein-coding transcripts. To further confirm that our cDNAs do not represent aberrant transcripts, we determined whether or not the cDNAs might be subject to nonsense-mediated decay (NMD) [35]. By definition, when an intron exists in more than 50 bp downstream of the stop codon, the transcript may be subject to NMD. We found that only 37 (6%) of the cDNAs might be subject to NMD.

We compared the CDSs which were deduced from our full-length cDNAs with the CDSs of the annotated gene models in a way similar to that in which we compared the 5'-end ESTs, and again found many inconsistencies. The discrepancies were, on average, (1) 14% at the nucleotide-level, (2) 36% at the exon-level, and (3) 41% at the gene-level. In 243 cases, there was at least one inconsistency in the CDS region of the annotated gene which altered the deduced amino acid sequence. The distribution of the altered amino acid lengths is shown in Figure 2c, again illustrating the need for full-length cDNA data in order to precisely annotate the genes.

### Detailed evaluation of inconsistent gene annotations

For the 586 full-length Tg cDNAs, out of 732 that correspond to unique annotated genes, we analysed the discrepancies in more detail. Namely we analyzed them with regard to their impact on the CDSs and observed that there were four major types of inconsistencies, involving either the translation start site or the termination site. We characterized them as follows:

(i) N-terminal inconsistency: only the first ATG site was inconsistent.

(ii) C-terminal inconsistency: only the translation terminal codon was inconsistent.

(iii) Internal inconsistency: both the first ATG site and the terminal codon were consistent, but there was at least one inconsistency in the body of the CDS.

(iv) Total inconsistency: both the first ATG site and the rest of the CDS were inconsistent.

**Figure 3:**
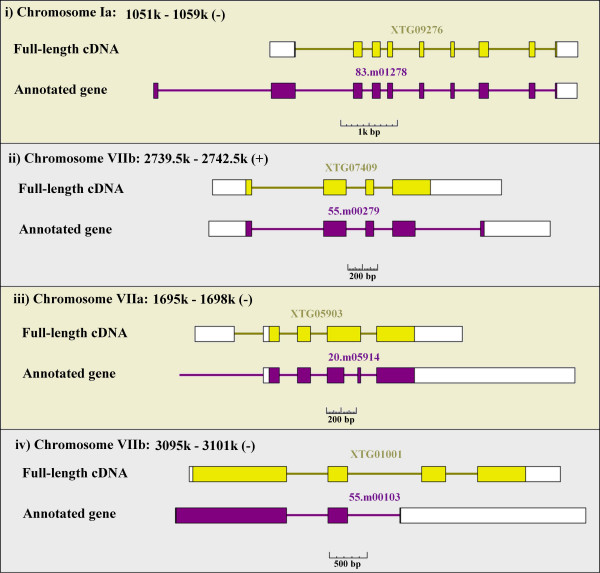
**Common patterns of inconsistencies in the CDS of the completely determined Toxoplasma full-length cDNAs**. Typical examples for categories i–iv as described in the text are shown. Yellow or purple boxes indicate the CDS.

Types (i) and (ii) comprised 65% of the inconsistencies (Table 4). For those of type (i), we found that most of the cDNAs had CDSs shorter than those of the annotated genes, with the first ATG site of the gene model usually being embedded in an upstream exon that does not exist in the actual cDNA (Figure 3). There is some concern that a shortened N-terminus represents an artifact due to erroneous cloning of a 5'-end-truncated cDNA by the oligo-capping method. However, we believe that such errors are infrequent because in most cases the 5'-ends of the cDNAs were covered by at least three independent cDNAs. For type (ii) inconsistencies, interestingly, a similar tendency was observed in the 3'-end and the C-terminal regions. Additional exons were predicted in the downstream regions of the gene models, so that the terminal codon in the cDNAs was bypassed due to questionable splicing. Similarly, we found that probable overprediction of exons was the major cause of inconsistencies for those of type (iii). In many cases, one exon in the cDNA was spuriously split into two in the gene model. These observations implicate that spurious prediction of extra exons in the annotated genes is the major cause of inconsistencies. The average number of exons was 3.4 and 3.9 for the cDNAs and the gene models, respectively; average overprediction in the latter was 0.5 exons per gene.

**Table 4:** Mismatch types in Toxoplasma

	Complete match	Type i	Type ii	Type iii	Type iv
Number of cDNAs	343	112	47	14	70
CDS (cDNA) average length (bp)	780	772	693	951	452
CDS (Gene model) average length (bp)	780	1,559	1,097	980	1,531
Average number of cDNA cluster members	9	3	13	6	5

For details about the categorization of types i–iv see text.

For the rest of the cases (type (iv)), the deduced amino acid sequences were inconsistent throughout the CDS. We were concerned that some of these inconsistencies may have been due to errors in the prediction of the CDS from the cDNA sequences, since two different strains, RH and ME49, were used for making the cDNAs and the genomic sequence, respectively. Sequence polymorphism (estimated to be 0.65% in this genome [36]) may have lead to some frame-shifts or base substitutions, thus changing the amino acid sequences and resulting in gene model inconsistency. When we substituted the original cDNA sequences for the mapped region sequences from the ME49 genome for CDS prediction, we found that 27% (10 of 37) of the re-predicted CDSs overlapped with CDSs of annotated genes to some extent at the deduced amino acid level. However, for the other 27 cases, the CDSs in the gene models did not overlap and will be analysed further.

### Consistency of full-length Toxoplasma cDNA transcripts

To examine the impact of the CDS changes, we analyzed the so-called functional annotations that were based on either the annotated gene models or the full-length cDNAs. We first looked for affected functional protein motifs using InterProScan to search the Pfam database [37]. The predicted functional motifs were found to be different in 43 cases, specifically, in 42 cases the motifs were found only in the annotated gene models but not in the cDNAs, indicating erroneous prediction, and in one case the motif was found in the cDNA but not in the annotated gene model (Additional file 6). There results indicate a tendency of gene prediction programs to assign exons in genomic regions if they encode amino acid sequences containing known motifs.

In addition, because we found many discrepancies in the N-terminal and C-terminal regions of the proteins (in which protein-sorting signals are often embedded), we examined the inconsistent motifs for subcellular protein sorting signals using PSORT [38] and found that changes in the deduced amino acid sequences resulted in altered subcellular localization of 213 cases (36%)(Additional file 7). Disturbingly, such errors in the gene models may seriously interfere with subsequent gene functional analyses.

### cDNAs non-overlapping with the annotated genes

We also analyzed 140 cDNAs that did not overlap with any of the annotated gene regions. Even in these cases, 131 cDNAs (94%) encoded proteins containing 50 or more amino acids (Additional file 7). When we examined the deduced amino acid sequences using BlastP (E-value < 1e-10) to check if there were any homologous proteins, significant hits were found in at least six cases. For the rest of the cases, we searched for protein functional domains using Pfam and found possible functional protein motifs in seven more cases (Additional file 8). To experimentally confirm the existence of these transcripts, we also performed RT-PCR (Additional files 9 and 10). In 118 of 135 clusters (87%), RT-PCR detected positive bands, suggesting there are actually transcripts at the corresponding regions. These results indicate that current gene annotations have overlooked a significant number of protein-coding genes that could have active biological roles. Interestingly, no clear CDSs were identified for nine cDNAs in our analysis. These cDNAs may correspond to non-protein-coding transcripts, many of which have been recently identified in parasite genomes [39], and may be important targets for further exploration of the apicomplexa transcriptome.

## Conclusion

In this paper, we describe the results of our analysis using 5'-end partial sequences from full-length cDNAs to systematically evaluate the current gene models for six species of apicomplexan parasites. Our results demonstrate that the current gene models need to be improved through annotations based on more intensive analyses using full-length cDNAs. While we could not determine the complete sequences for all of the cDNAs, except for Toxoplasma, the remaining sequence information is just as critical to complete. Emerging technologies such as next-generation sequencers may speed up such analyses and reduce their overall cost [40]. Further integration of the pre-existing data, such as publicly available ESTs and peptide sequences, is also important. The integration of various experimental approaches and data will lead to increasingly accurate genome annotations and thus provide a solid basis for further research on apicomplexan parasites.

## Methods

### Generation of the 5'-EST sequence data

We constructed full-length cDNA libraries using the oligo-capping method [25,41]. The experimental procedures for library construction are described in the references, along with details about each of the libraries (Additional file 11). Glycerol stocks were generated by selecting approximately 15,000 clones from each library which were sequenced using ABI3730 sequencers following standard protocols for sequencing analysis.

After trimming the vector sequences and low quality regions, we mapped the cDNA sequences onto their corresponding genomic sequences using SIM4 [38] with default parameters. The mapping results were then filtered for those with sequence identity >= 0.95, coverage >= 200 bp, and having the first base map to a unique genome location. Redundantly mapped cDNAs with similar mapping scores were removed from the dataset. The filtered partial cDNA sequences were deposited in the DNA Data Bank of Japan [DDBJ: DK887268–DK936566]. The physical cDNA clones will soon be deposited at MR4.

### Generation of the complete cDNA sequence data for Toxoplasma

Non-redundant cDNAs from the 5'-EST dataset were selected for complete sequencing analysis using the primer-walking method. The sequences obtained were then assembled using two different methods: a genome-sequence-independent method and a genome-sequence-guided approach. The genome mappings for the latter method were performed in a similar way to that used for the EST mapping (except for the additional filtering of the first base requirement). For cDNA assembly, a standard assembler program, Phrap [42], was used.

### Comparison of the cDNAs and the annotated gene models

The latest gene annotation models were downloaded from the PlasmoDB [12], CryptDB [15] and ToxoDB datasets[18] (pf_rel5.4, pv_rel5.4, py_rel5.4, pb_rel5.4, cp_rel3.7 and tg_rel4.3). Information about the genomic coordinates of the exons and the positions of the protein-coding regions, as well as other annotation information, was extracted from the corresponding GFF files. The mapping coordinates for all species, except Tg, were extracted from the same GFF files. For Tg, the mapping procedure was as described in the mapping of the partial cDNAs. The obtained genomic coordinates were compared between the cDNAs and the annotated gene models, and an annotated gene model was considered to correspond to a cDNA when at least a part of one its exon was identical to a region of the cDNA. All of the alignments are available on our website [27].

The longest ORFs were assigned as the protein coding regions for the complete Tg cDNA sequences. The amino acid sequences for the annotated gene models were extracted from the information in the ToxoDB GFF files. The CDS overlaps were evaluated again at the genomic sequence level, with each set of evaluation data for the gene annotations being based on manually inspected cDNA evidence using the graphical interface of our Full-parasites and Comparasite databases (also see Additional file 12) [29].

### Functional annotation of the cDNAs

The deduced amino acid sequences were searched for functional protein motifs using InterProScan [43] and the Pfam database (InterPro 16.1) with default parameters. The annotated gene models were similarly searched for functional motifs, even though in some cases the annotation information was already available in the public dataset. Subcellular localization was similarly predicted using "PSORT animal" [38] with default parameters.

### Analysis of the TSSs and 5'-UTRs

For analysis of the TSSs and 5'-UTRs, cDNAs that corresponded to the same annotated gene were clustered. The average number of clone members was calculated as the average number of cDNAs belonging to the particular annotated gene. The average number of TSS positions was calculated as the number of independent genomic positions to which the 5'-ends of the cDNAs mapped. The average TSS distribution was calculated as the average standard deviation from the average TSS position for each cluster. The average length of the 5'-UTRs was calculated as the distance between the TSSs of the cDNAs and the CDS start of the annotated gene models, and the distribution of the 5'-UTR length was calculated accordingly. In some cases the ATG start codon was not located in the first exon, i.e. the 5'-UTR contained one or more introns; such cases were calculated and recorded. Correlation between the 5'-UTR length and the Gene Ontology term as defined by InterProScan was evaluated by the Wilcoxon rank test.

### RT-PCR analysis of the non-overlapping cDNAs in Toxoplasma

For experimental validation of the non-overlapping cDNAs in Toxoplasma, real-time RT-PCR was performed using the 7900HT (ABI) following standard protocols. The PCR primer sequences are listed in Additional file 10. Total RNA was isolated during the Toxoplasma tachyzoite stage and first-strand cDNA was synthesized using an oligo-dT adaptor primer (5'-GCG GCT GAA GAC GGC CTA TGT GGC CTT TTT TTT TTT TTT TTT-3'). For RT-PCR, 1 ng of first-strand cDNA was used. For the negative control, mock first-strand cDNA synthesis was performed without reverse transcriptase. The results were considered positive when there was at least an 8-fold difference in amplification versus the negative control (3-cycle difference in the Ct (cut-off of threshold) value) and a clear band was obtained with agarose gel electrophoresis.

## Abbreviations

bp: base pair(s); cDNA: complementary DNA(s); SAGE: serial analysis of gene expression; TSS: transcriptional start site(s); CDS: coding sequence(s); EST: expressed sequence tag(s); ORF: open reading frame(s); PCR: polymerase chain reaction; UTR: untranslated region(s); NMD: nonsense-mediated decay; Pf: *Plasmodium falciparum*; Pv: *Plasmodium vivax*; Py: *Plasmodium yoelii*; Pb: *Plasmodium berghei*; Cp: *Cryptosporidium parvum*; Tg: *Toxoplasma gondii*.

## Authors' contributions

HW performed the sequence alignments and statistical analysis, and drafted the manuscript. YS conceived the study, participated in the design and coordination of the project, and helped to draft the manuscript. MS participated in library construction with the aid of SS. JW coordinated the study and supplied the materials.

## Supplementary Material

Additional file 1 **Statistics for the oligo-capped cDNA clones**. Statistics for the oligo-capped cDNA clones are shown. Pf, Pv, Py, Pb, Cp and Tg indicate *Plasmodium falciparum*, *Plasmodium vivax*, *Plasmodium yoelii*, *Plasmodium berghei*, *Cryptosporidium parvum *and *Toxoplasma gondii*, respectively. Click here for file

Additional file 2 **Details of 5'-end-one-pass cDNA analysis results**. Information about mapped positions of the cDNAs, corresponding annotated gene models, predicted protein motifs, and GO categories are shown. For the cDNAs which did not correspond to any annotated gene, the columns for annotated gene information are blank. Genomic versions used in this study are pf_rel5.4, pv_rel5.4, py_rel5.4, pb_rel5.4, cp_rel3.7 and tg_rel4.3, respectively. Column A: Type of hit for annotated gene. One asterisk (*) indicates no hit to annotated gene. Two asterisks (**) indicate a hit to more than one annotated gene. Column C: Cluster ID [internal use]. Column E: Nucleotide length of one-pass read sequences. Columns F-I: Mapped position of cDNA. Start/End simply represents the order of genomic coordinates, therefore the TSS is at the "start" when the genomic strand is "+" and at the "end" when it is "-". Columns J-O: Information on annotated gene models corresponding to the cDNA. "Putative UTR length" is the distance between the cDNA start (TSS) and the CDS start of the annotated gene model. Click here for file

Additional file 3 **Statistics for apicomplexan genomes and annotated gene models**. ND: not done. NA: not applicable. Click here for file

Additional file 4 **Example of erroneous annotation**. PF13_0024 (annotated gene of Pf) is separated into two annotated genes in Pb, possibly because PB000757.01.0 is on contig PB000757.01.0 and PB000975.00.0 is on contig PB_RP1621. Click here for file

Additional file 5 **Results of Wilcoxon Rank Sum Test**. Result of "corresponding to ribosome" is listed for each species. Click here for file

Additional file 6 **Affected Pfam motifs**. Affected Pfam motifs are listed. InterProScan was used to search the Pfam database for protein motifs. Click here for file

Additional file 7 **Details of Tg full-length sequence analysis results**. Information about mapped positions, corresponding annotated genes, predicted protein motifs and GO categories, blastP hits and subcellular localization are shown. Column A: Type of hit for annotated gene. One asterisk (*) indicates no hit to annotated gene. Two asterisks (**) indicate a hit to more than one annotated gene. Column D: Amino acids sequence as deduced from the nucleotide sequence of the longest open reading frame (ORF). Columns G-J: Mapped position of the cDNA. Columns M-R: Functional annotation from the deduced amino acid sequence based on our cDNA data. Columns T-AB: Functional annotation using the amino acid sequence deduced from the annotated CDS. Columns K-L: Position of the CDS identified as the longest ORF. Columns M and V: Results of transmembrane domain search using SOSUI [44]. Columns N and W: Results of subcellular localization site search using PSORT [38]. Columns Q, R, AA and AB: Results of protein domain search using InterProScan [43]. Columns T-AB: Information about the annotated gene model(s) which correspond to the clone. Column X: Marked when the subcellular localization predicted by PSORT was different between Column M (cDNA) and V (annotated gene). Click here for file

Additional file 8 **Results of BlastP and Pfam motif searches**. List of affected BlastP and Pfam motifs for Tg full-length cDNAs which do not overlap annotated genes. Click here for file

Additional file 9 **Real-time RT-PCR evidence for transcription**. Examples of the results for real-time RT-PCR. IDs of the cDNAs are shown in the margin. (+): with reverse transcriptase; (-) without reverse transcriptase. Gray box: negative (For more details, See Additional file 4). Click here for file

Additional file 10 **RT-PCR result details**. UD: undetermined. ND: not done. NA: not applicable. UD, ND and NA are all shaded. Ct value (PCR cycle value for threshold) more than 35.0 is also shaded. Click here for file

Additional file 11 **Culture and development stage information**. Information about strains, development stages and cultures are shown. Click here for file

Additional file 12 **Database search for cDNA-genome alignment.** Click here for file
