# Supplementary figures and images for: Inconsistencies of genome annotations in apicomplexan parasites revealed by 5'-end-one-pass and full-length sequences of oligo-capped cDNAs
Source: BMC Genomics. 2009 Jul 15;10:312. doi: 10.1186/1471-2164-10-312 (PMC2722674; doi:10.1186/1471-2164-10-312)

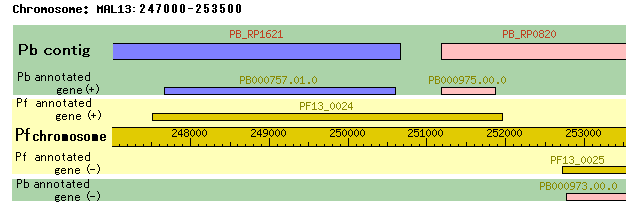

Supplement: Additional file 4 — Example of erroneous annotation. PF13_0024 (annotated gene of Pf) is separated into two annotated genes in Pb, possibly because PB000757.01.0 is on contig PB000757.01.0 and PB000975.00.0 is on contig PB_RP1621. [file 1471-2164-10-312-S4.png]

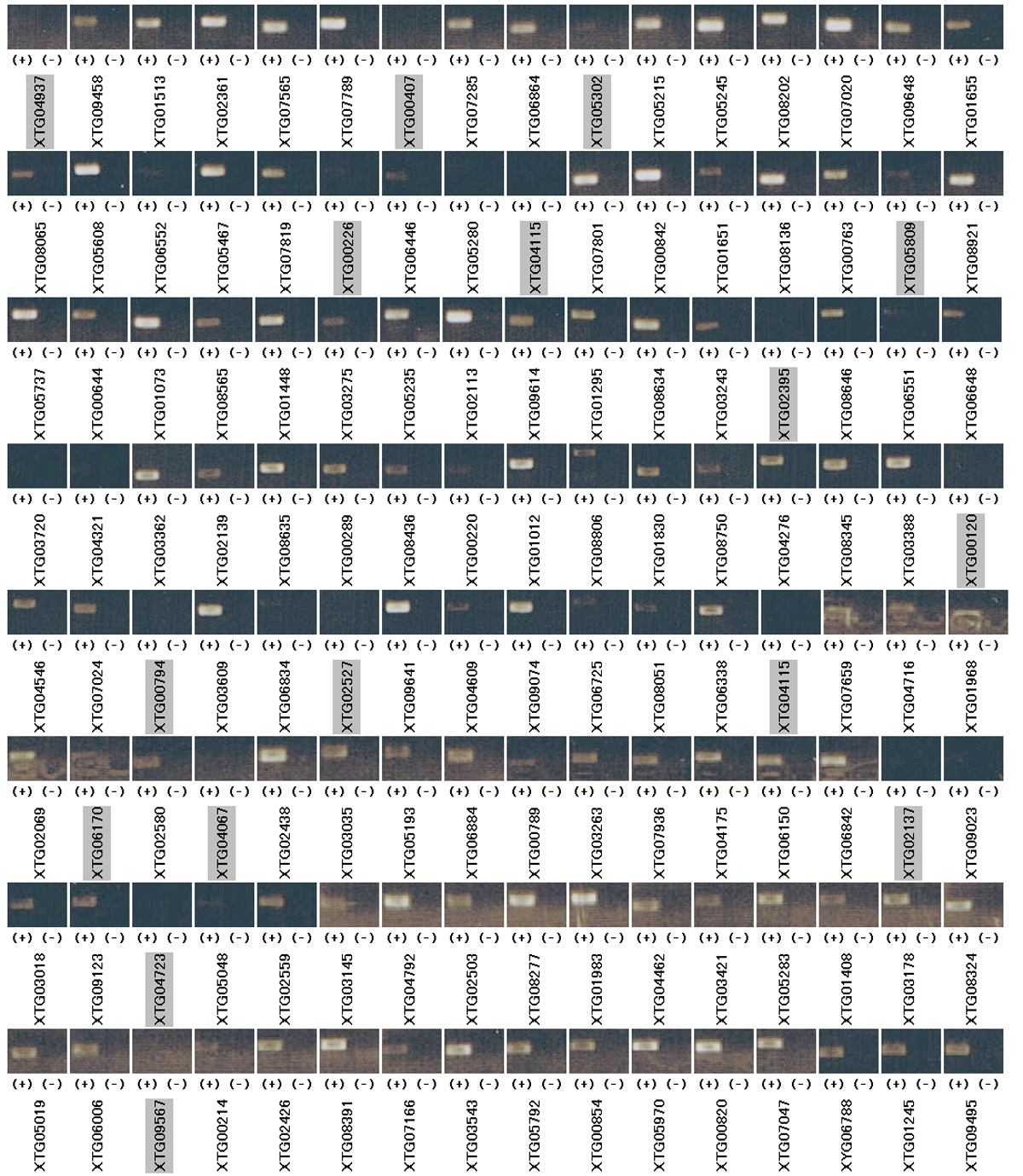

Supplement: Additional file 9 — Real-time RT-PCR evidence for transcription. Examples of the results for real-time RT-PCR. IDs of the cDNAs are shown in the margin. (+): with reverse transcriptase; (-) without reverse transcriptase. Gray box: negative (For more details, See Additional file 4). [file 1471-2164-10-312-S9.jpeg]

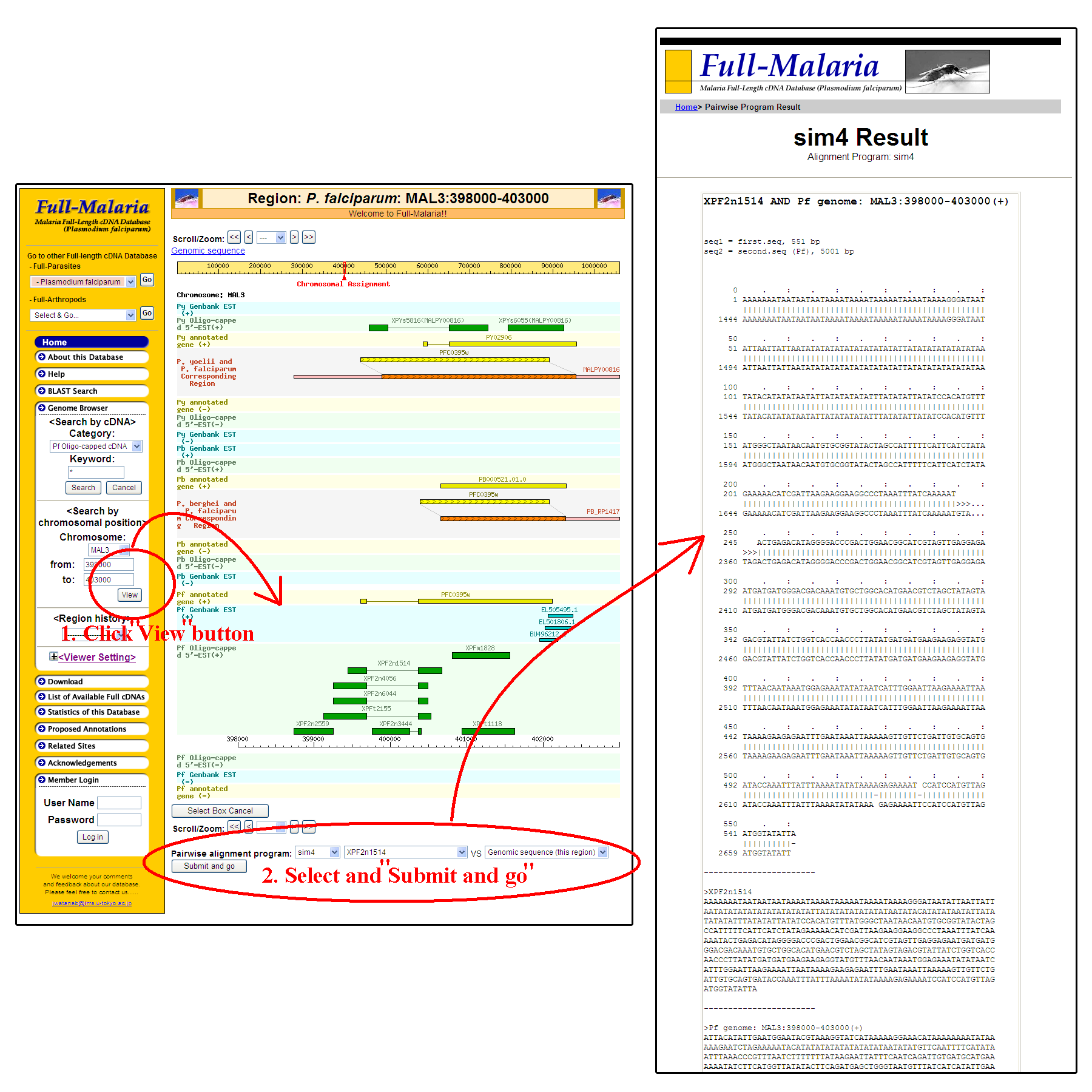

Supplement: Additional file 12 — Database search for cDNA-genome alignment. [file 1471-2164-10-312-S12.png]
